# Advanced Dissection Lab for Neuroanatomy Training

**DOI:** 10.3389/fnana.2021.778122

**Published:** 2022-01-05

**Authors:** Giuseppe La Rocca, Edoardo Mazzucchi, Fabrizio Pignotti, Gianluca Galieri, Pierluigi Rinaldi, Giovanni Sabatino

**Affiliations:** ^1^Department of Neurosurgery, Mater Olbia Hospital, Olbia, Italy; ^2^Institute of Neurosurgery, Istituto di Ricovero e Cura a Carattere Scientifico Fondazione Policlinico Universitario Agostino Gemelli, Catholic University, Rome, Italy; ^3^Neurosurgical Training Center and Brain Research – Mater Olbia Hospital, Olbia, Italy; ^4^Unit of Radiology, Mater Olbia Hospital, Olbia, Italy

**Keywords:** neuroanatomy, brain dissection, cadaver lab, neuroanatomy training, neurosurgeon teaching

## Introduction

Contemporary neurosurgical training can take advantage of innovative teaching methods that have been offered by the significant and continuous development of surgical instruments, techniques, and communication strategies (Signorelli, 2019).

Neuroanatomical dissection is a fundamental step for neurosurgical training. However, the availability of specimens for neuroanatomical dissection may be limited or expensive if the specimens have to be imported from other countries. Thus, we think that a way to fully exploit the educational potential of neurosurgical dissection is to employ multiple technological tools, which assist the trainees during the dissection and create the possibility to collect photographs, videos, and radiologic images. The acquired information may be analysed after dissection by the trainee who is performing the dissection, but also larger groups of students can benefit from this considerable amount of data.

The “Neurosurgical Training Center and Brain Research” in Mater Olbia Hospital meets this necessity and offers the best dissection experience in a surgical theatre scenario.

## Materials and Methods

The “Neurosurgical Training Center and Brain Research” was set up under the internal Institutional protocol number 338, on 23 February 2021. Two cadaver heads injected with coloured silicone (see Sanan et al., 1999) with no brain injury as cause of death were provided. Both specimens were imported from another country, respecting all local and international laws. The specimens were stored in a bucket at 4°C in 10 L of solution: 69% water, 13.8% ethanol, 8.6% glycerine, and 8.6% of 10% formaldehyde (Fisher Chemical^®^, Hampton, NH, USA).

Preoperative CT scans (GE Healthcare, Little Chalfont, UK, Revolution™ EVO CT System, version Revolution Evo 1.1, 64 slices) and MRIs [GE Healthcare, SIGNA ARTIST MRI Machine, 1.5 Tesla (T), model A670060237] with neuronavigation cuts were performed.

Intraoperative tools were an AIRO^®^ iCT scan (Mobius Imaging LLC, AIRO, version 2.1.0.2) and BrainLab navigation software (AIRO^®^ version 2.1.0.2 BrainLab, Munich, Germany), and a BK^®^ ultrasound (bk5000-01 Ultrasound System, BK Medical Aps, Mileparken, Denmark).

**Figure 1:**
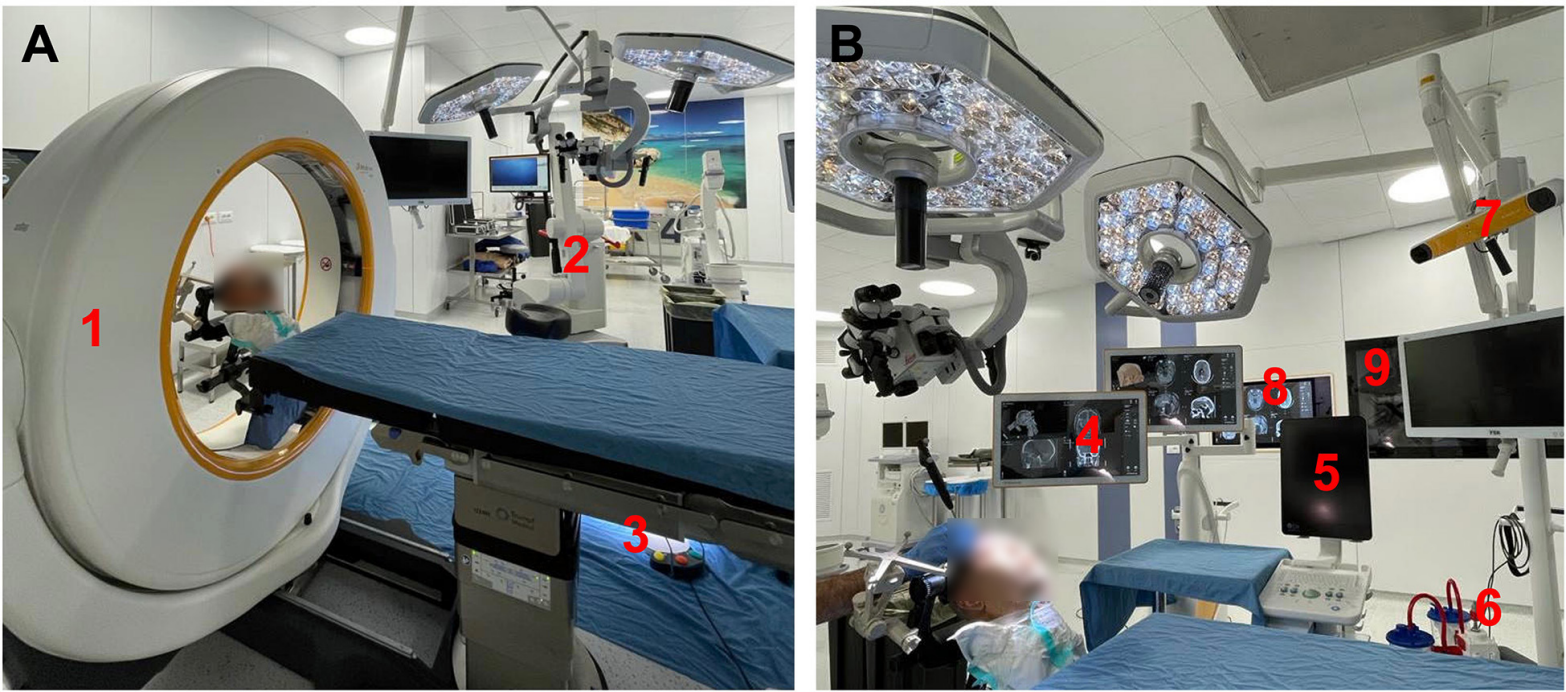
**(A)** (1) Pre-dissection setting intra-operative CT (AIRO), (2) Leica microscope, (3) High Speed Drill foot pedal. **(B)** Dissection setting: (4) MRI merged with CT scan for neuronavigation, (5) BK ultrasound, (6) suction, (7) Neuronavigator antenna, (8) BrainLab software, and (9) high-resolution student viewing display.

The specimen was fixed with a three-pin Mayfield holder, and the neuroanatomical dissection with microsurgical instruments was performed under the magnification of a Leica^®^ microscope (Leica M530 OHX, Leica Microsystems, Heerbrugg, Schweiz). Olympus^®^ (Olympus Visera 4K UHD OTV-S400 and Olympus Visera 4K UHD CLV-S400, Olympus Medical System Corp., Tokyo, Japan) endoscopic tools were available for endoscopic-assisted dissection (Figure 1).

The Medtronic^®^ High Speed Drill (Medtronic IPX1, IPC System Medtronic Xomed Inc., Jacksonville, FL, USA) and Sonopet^®^ Ultrasonic Aspirator (Stryker Neuro Spine ENT, Kalamazoo, MI, USA) were used for craniotomy and bone removal.

Pictures and videos were recorded with the microscope system and stored for educational purposes.

Dissection was actively performed by residents in neurosurgery, with the help of expert neurosurgeons. Medical students assisted the dissection sessions. The aim of dissection was both a standard anatomical dissection and a surgical approach simulation. The MRI and CT images were fused with the help of the neuronavigation software, similarly to a standard neurosurgical intervention. This allows the identification of several anatomical structures in radiological images and elaboration of 3-dimensional reconstructions (de Notaris et al., 2013, 2014).

The trainees had to put the head in the surgical position and perform a pre-dissection CT scan to be integrated with the set of images for neuronavigation, just like before a surgical intervention, to allow the use of neuronavigation. The trajectory and the craniotomy could be chosen and simulated in the neuronavigation software. Any time they wanted, the trainees could verify the position of the anatomical structure they had dissected by using the neuronavigation pointer. Moreover, an “intra-operative” CT scan could be performed at any step of dissection, for example, to verify the amount of bone removed. At any stage, photographs and videos were captured and coupled with the position of the instrument or of the microscope (acquired thanks to the neuronavigation system).

## Discussion

Neuroanatomy is the initial step of all neurosurgeons' education, and it is fundamental at every training level. It is particularly relevant for a more sophisticated understanding of the best surgical strategy (Sanan et al., 1999; de Notaris et al., 2013; Signorelli, 2019). The deepest knowledge can be achieved, in our opinion, only by coupling in-depth theoretical study with applied experience during anatomical dissection or surgical activity (Ghosh, 2017). On the other hand, the availability of specimens and of adequate facilities to regularly perform dissection is very limited in some geographical areas. A possible solution to overcome this relevant problem in neurosurgical education is the constitution of a laboratory for neuro-anatomical dissection, which allows the use of an armamentarium of technologically advanced tools to maximise the quality of dissection and, at the same time, allows post-dissection review of the data (Rodriguez Rubio et al., 2019).

In our experience, there are different kinds of problems to fix before setting a neuroanatomic laboratory for research or teaching purpose.

First, the current Italian legislation makes it very difficult to use cadavers for anatomo-surgical dissection (Signorelli, 2019). For these reasons, only in the last years, centres for body collections and programmes for body donations have been developed (De Caro et al., 2009).

Second, in this scenario, universities, or anatomical dissection centres, could get specimens from a private company outside Italy. It is (1) time-consuming, (2) expensive, and (3) it has several bureaucratic steps that need to be sorted out. After delivery, specimens should be kept in proper solution at 4°C when not used for dissection.

Third, CT scan, MRI, and all intraoperative tools require skilled teachers and, at the same time, need preparation time and precious resources from the centre.

In our local experience, the deep conviction of the need for constant improvement of neuroanatomical knowledge of the neurosurgical department met the fruitful collaboration of the Radiology and Laboratory Medicine departments. This cooperation between different departments put forward the basis for the establishment of the Laboratory with limited amount of funds, mainly thanks to unpaid voluntary work.

Before starting dissection, the trainees need to study anatomy in the textbook and then draft a procedure to perform on the specimens. We think that neuroanatomical dissection is more beneficial for a neurosurgical resident if it explores a subject which has implications in neurosurgical activity. Moreover, if the trainee follows a predefined schedule, the same head can be more extensively exploited without “wasting” any possibility of learning that is avoiding, for example, any damage to anatomical structures that can be useful for other projects.

This is one of the hardest steps, but it is of crucial importance. In this phase, the trainees do not have a precise idea of what kind of dissection to perform because they have not mastered neuroanatomy and every idea seems to be already thoroughly examined in literature.

At the end of this pre-dissection period, the project is prepared, and the specimen is ready for dissection. At the moment, we are encouraging the participation of residents in neurosurgery for a 1–3-month dissection period.

As other colleagues reported (Kobayashi et al., 2021), first attempts are always quite challenging: a study of pertinent anatomy, dissection, and data collection both for research and for educational purposes are difficult and need time (Matsushima et al., 2018; Leonel et al., 2021). Up to today, Italian residents have difficulties in accessing dissection courses because they are expensive, often not easy to reach, and in the classic 2-day course, there is too much content to get through.

Dissection is a work of handicraftsmanship that should be approached without pressure. Day-by-day expertise and competence will grow quickly, and advanced complexity dissections can be tried.

At the end of the dissection program, anatomical studies can be followed by clinical application (La Rocca et al., 2017, 2021) or can be used as a test of clinical insight, such as the comparison between different approaches for the same target (Sturiale et al., 2017; La Rocca et al., 2018).

The multidisciplinary work is precious and fundamental because different expertise is needed to set up a dissection program.

The aid of pre-operative CT scans and MRI, intraoperative CT scans with BrainLab navigation software, high-definition surgical microscopes and endoscopes, and microsurgical instruments together with high-definition image data acquisition and records can strongly enhance the dissection experience (de Notaris et al., 2013). This set of instruments allows a precise pre-dissection planning of the approach to be performed, guides dissection with the possibility of a continuous update of the actual anatomic situation (by performing the “intra-operative” CT scan), and enables both the trainees and other students to extensively review each phase of the dissection thanks to the variously collected data: photographs, videos, CT scan images, neuronavigated points, and trajectories.

## Conclusion

Neuroanatomy is fundamental in neurosurgical growth, and it remains a critical frontier in our field. Promotion of donation plans could allow universities to have more specimens and facilitate trainees to experience dissection work. A well-designed dissection period could be a unique training opportunity for residents and young specialists. The presence of multiple technologically advanced tools in the dissection laboratory may help the trainees get the best learning experience despite the limited availability of specimens.

## Author Contributions

GL and GS: conception and design of the study and final approval of the version to be submitted. EM, FP, GG, and PR: acquisition of data. GL and EM: drafting the article or revising it critically for important intellectual content. All authors contributed to the article and approved the submitted version.

## Conflict of Interest

The authors declare that the research was conducted in the absence of any commercial or financial relationships that could be construed as a potential conflict of interest.

## Publisher's Note

All claims expressed in this article are solely those of the authors and do not necessarily represent those of their affiliated organizations, or those of the publisher, the editors and the reviewers. Any product that may be evaluated in this article, or claim that may be made by its manufacturer, is not guaranteed or endorsed by the publisher.
